# Lack of mitochondrial MutS homolog 1 in *Toxoplasma gondii* disrupts maintenance and fidelity of mitochondrial DNA and reveals metabolic plasticity

**DOI:** 10.1371/journal.pone.0188040

**Published:** 2017-11-15

**Authors:** Tamila Garbuz, Gustavo Arrizabalaga

**Affiliations:** 1 Department of Pharmacology and Toxicology, Indiana University School of Medicine, Indianapolis, Indiana, United States of America; 2 Department of Microbiology and Immunology, Indiana University School of Medicine, Indianapolis, Indiana, United States of America; University at Buffalo, UNITED STATES

## Abstract

The importance of maintaining the fidelity of the mitochondrial genome is underscored by the presence of various repair pathways within this organelle. Presumably, the repair of mitochondrial DNA would be of particular importance in organisms that possess only a single mitochondrion, like the human pathogens *Plasmodium falciparum* and *Toxoplasma gondii*. Understanding the machinery that maintains mitochondrial DNA in these parasites is of particular relevance, as mitochondrial function is a validated and effective target for anti-parasitic drugs. We previously determined that the *Toxoplasma* MutS homolog TgMSH1 localizes to the mitochondrion. MutS homologs are key components of the nuclear mismatch repair system in mammalian cells, and both yeast and plants possess MutS homologs that localize to the mitochondria where they regulate DNA stability. Here we show that the lack of TgMSH1 results in accumulation of single nucleotide variations in mitochondrial DNA and a reduction in mitochondrial DNA content. Additionally, parasites lacking TgMSH1 function can survive treatment with the cytochrome b inhibitor atovaquone. While the *Tgmsh1* knockout strain has several missense mutations in cytochrome b, none affect amino acids known to be determinants of atovaquone sensitivity and atovaquone is still able to inhibit electron transport in the *Tgmsh1* mutants. Furthermore, culture of *Tgmsh1* mutant in the presence atovaquone leads to parasites with enhanced atovaquone resistance and complete shutdown of respiration. Thus, parasites lacking TgMSH1 overcome the disruption of mitochondrial DNA by adapting their physiology allowing them to forgo the need for oxidative phosphorylation. Consistent with this idea, the *Tgmsh1* mutant is resistant to mitochondrial inhibitors with diverse targets and exhibits reduced ability to grow in the absence of glucose. This work shows TgMSH1 as critical for the maintenance and fidelity of the mitochondrial DNA in *Toxoplasma*, reveals a novel mechanism for atovaquone resistance, and exposes the physiological plasticity of this important human pathogen.

## Introduction

*Toxoplasma gondii* is a pervasive obligate intracellular protozoan parasite known to infect human and all other warm-blooded animals. Infection with *Toxoplasma* is usually asymptomatic in most healthy individuals as a robust immune response is able to exert pressure on the rapidly dividing parasites and forcing them into a dormant form, thereby limiting the tissue destruction caused by the parasite. However, patients with a compromised immune system, such as transplant recipients receiving immunosuppressant therapies and patients with AIDS, are at a great risk for developing life-threatening illness as a consequence of the parasite’s unchecked growth [1]. Additionally, infection with the parasite during pregnancy can cause severe neurological defects or even death of the developing fetus. First line therapy against acute toxoplasmosis consists of the antifolate pyrimethamine, in combination with sulphadiazine. Nonetheless, these compounds have no effect on the dormant encysted form of the parasite but there are significant side effects associated with their use as they can affect the host’s folate synthesis. Considering that approximately a third of the world’s population is infected with this pathogen and given the significant patient population with compromised immune systems, infection with *Toxoplasma* is a serious public health concern—therefore, the discovery and study of alternative therapies is a priority [2].

Work on *Toxoplasma* and other parasites of the phylum Apicomplexa, such as the causative agent of malaria, *Plasmodium falciparum*, has pinpointed their single mitochondrion as an ideal drug target due to its essential function in the parasite’s life cycle and more importantly its genetic and structural divergence from the host mitochondria [3–5]. Current drugs that are known to target the mitochondrion of these parasites include atovaquone, myxothiazol, antimycin A and clopidol [6–9]. Of these drugs, atovaquone has been used therapeutically to treat human pathogens such as, *Plasmodium*, *Toxoplasma*, *Babesia* and *Pneumocystis*. Co-treatment with proguanil is often required to mitigate some of the limitations associated with mono-atovaquone treatment, such as bioavailability and resistance [10].

While the importance of *Toxoplasma’*s mitochondrion for parasite survival and its validity as a drug target is well established, much of its biology remains to be elucidated. *Toxoplasma* has a single mitochondrion, which like that of most protozoa is a double-membraned organelle with tubular rather than plate-like cristae. One major barrier in the study of the mitochondrion in *Toxoplasma*, is the fact that its genome has yet to be fully characterized and sequenced, primarily due to the presence of fragments of mitochondrial genes inserted into the nuclear genome [11]. Based on the mitochondrial genomes found in other apicomplexans, such as *P*. *falciparum*, the *Toxoplasma* mitochondrial genome is thought to have three gene products, cytochrome b (*Cyb*), cytochrome c oxidase I (*Cox1*), and cytochrome c oxidase III (*Cox3*), along with interspersed rRNA elements [12]. Thus, compared to the 13 genes found in the mammalian mitochondrial genome, the apicomplexan mitochondrial genome is significantly reduced. Nonetheless, the importance of maintaining the function of the few genes encoded in the apicomplexan mitochondria is underscored by the fact that inhibition of cytochrome b by atovaquone is lethal to the parasite [9]. However, as of yet, little is known about how the mitochondrial DNA of apicomplexan parasites is maintained and repaired.

As several of the proteins critical for mitochondrial function are encoded within the mitochondrial DNA (mtDNA), maintaining its integrity and fidelity is important for cell survival. While initially it was thought that the mitochondria did not possess DNA damage repair activity, it is now clear that mtDNA is heavily monitored and modified by multiple repair pathways. Mechanisms of DNA repair in the mitochondria parallel those present in the nucleus, including base excision repair (BER), single-strand break repair (SSB), and mismatch repair (MMR) [13]. Both BER and SSB in the mitochondria are executed by the same proteins that, as a result of dual localization or alternative splicing, also drive nuclear DNA repair. By contrast, nuclear MMR proteins are not detected in mammalian mitochondria [14,15] and genetic disruption of nuclear MMR proteins does not affect mtDNA stability [16], indicating that the MMR systems in the nucleus and mitochondria are distinct from each other. In the nucleus, the key components of the MMR machinery are MutS homologue 2 (MSH2), which forms heterodimeric complexes with MutL homolog 1 and either MSH6 or MSH3. In mammalian mitochondria it has been suggested that the Y-box binding protein, YB-1, is responsible for recognizing DNA mismatches and, accordingly, its depletion causes accumulation of mutations in mtDNA [15]. However, the functional partners of YB-1 are not yet known. By contrast to mammalian cells, yeast possess a MutS homolog, MSH1, that localizes exclusively to the mitochondria. Importantly, MSH1 disruption results in severe mtDNA instability [17–19]. Similarly, plants encode for a MSH1 that localizes to the mitochondria and regulates substoichimetric shifting [20].

*Toxoplasma* appears to have full complements of the nuclear BER and SSB factors, however, it is not known whether these proteins also localize and function in the mitochondrion. As to mitochondrial MMR, *Toxoplasma* has a clear MSH1 homolog, TgMSH1 [21], but no clear equivalent to YB-1. Thus, if mismatch repair occurs in the *Toxoplasma* mitochondria it is likely to be more akin to that of yeast and plants rather than mammalian cells. We previously reported that TgMSH1 localizes to the mitochondrion of *Toxoplasma* and that its disruption renders the parasite resistant to the antiparasitic ionophore, monensin, and to the alkylating agent, methylnitrosourea. This resistance phenotype is directly due to the lack of TgMSH1 rather than a mutator effect, as sensitivity is restored by complementation with a wildtype copy of the gene. In higher eukaryotes, nuclear mismatch repair enzymes are also known to directly signal cell cycle arrest and apoptosis in response to cytotoxic levels of DNA damage [21]. Accordingly, we hypothesized that monensin and other drugs that directly or indirectly affect the mitochondrion elicit a signaling cascade that is dependent on TgMSH1. Consistent with this model, we have shown that monensin causes cell cycle arrest and autophagic death in a TgMSH1 dependent manner [22,23].

However, whether TgMSH1 functions as a bona fide DNA repair enzyme within the mitochondrion of *Toxoplasma* has yet to be explored. MutS and MSH proteins typically contain five structural domains of which domains I and IV interact with DNA and domain V contains an ATPase and dimerization interface [24,25]. Domains II and III form a connector domain and the core domain respectively [24,26]. Interestingly, TgMSH-1 contains domains I and V, but appears to lack domains II, III and IV. While this is an unusual arrangement for MSHs, the MSH-1 protein in *Arabidopsis thaliana* (AtMSH-1), which also localizes to the mitochondria, shares a similar domain organization with TgMSH-1 at the sequence level [20]. In addition, like AtMSH-1, TgMSH1 includes a C-terminal GIY-YIG endonuclease domain, which in the protein from *A*. *thaliana* is necessary for protein function [21,27]. Within the plant mitochondria, AtMSH-1 is thought to play a role in the suppression of recombination or amplification of mitochondrial DNA molecules [20]. Thus, given the sequence and domain arrangement similarities, it is plausible that TgMSH1 has parallel functions in *Toxoplasma*. Here we show that indeed TgMSH1 is required to maintain the fidelity and stability of the *Toxoplasma* mitochondrial genome. Furthermore, we show that the parasite adapts to the accumulation of mutations within the mitochondrial DNA by reversibly shutting down mitochondrial function, revealing the metabolic plasticity of this important human pathogen.

## Results

### Strain lacking TgMSH1 function accumulate mutations in mitochondrial DNA

**Fig 1:**
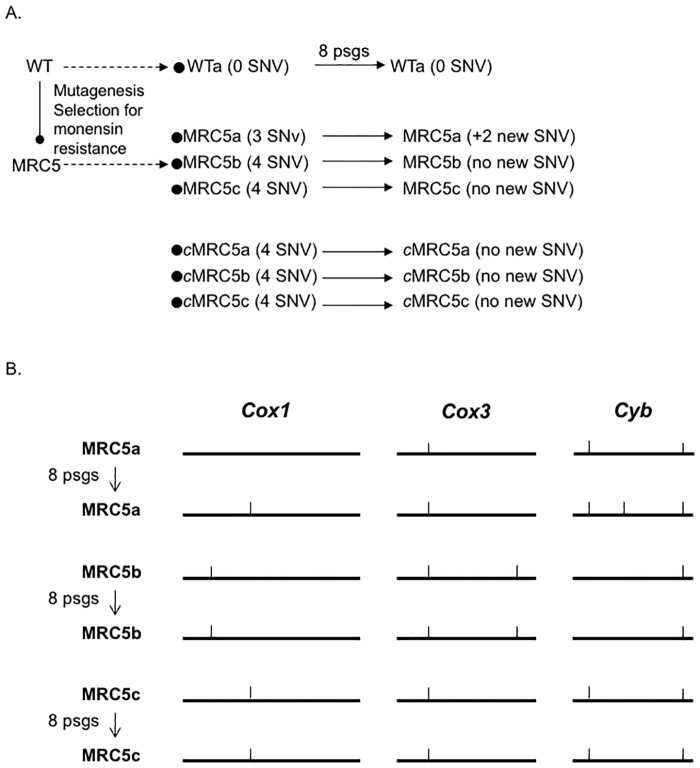
Monitoring of mitochondrial DNA in parasites lacking TgMSH1. A. Sequences of three mitochondrial fragments were monitored in clones of the parental (WT clone a), *Tgmsh1* mutant (MRC5 clones a, b and c), and complemented mutant (*c*MRC5 clones a, b, and c) strains after serial passage in culture. Horizontal arrows indicate passages in culture; solid ones indicate a known specific number of passages (psgs), which is indicated above. Black circle represents the establishment of clones of the particular strain by limiting dilution. The number of single nucleotide variants (SNV) indicated is in respect to the sequence of the parental strain. B. Schematics depict the emergence and relative position of SNVs in the MRC5 strain within the three fragments of mtDNA monitored, *Cox1*, *Cox3*, and *Cyb*. Tick marks represent relative position of SNV.

In yeast, the lack of the MutS homolog Msh1p leads to an increased rate of spontaneous point mutations in the mitochondrial DNA (mtDNA) [17]. Accordingly, we set out to investigate whether our *Tgmsh1* mutant strain, MRC5, accumulated mutations in its mtDNA during normal tissue culture maintenance. Using a putative assembly of the *Toxoplasma* mtDNA (Dr. Jessie Kissinger, personal communication) we designed primers that specifically amplify small fragments of the mitochondrial genes *Cox1* (339 bp), C*ox3* (181 bp) and *Cyb* (176 bp) and that should be unable to amplify fragments of nuclear DNA (nDNA) (S1 Table and S1 Fig). We sequenced these fragments from fresh clones established from the parental (WT), the mutant (MRC5), and complemented (*c*MRC5) strains, all of which had been maintained in culture for a prolonged period (approximately 2 years). While the parental clone exhibited no single nucleotide variations (SNV) in respect to the sequence of each of the genes deduced from the putative assembly and the genome database, a total of 6 unique SNVs were found within those fragments from the mutant clones (3 SNVs in clone MRC5a, 4 in MRC5b and 4 in MRC5c, Fig 1). Interestingly, each the three clones established from the complemented strain had the same 4 SNVs within the small fragments sequenced (Fig 1A). Since the complemented strain was generated using the MRC5 mutant strain that had been in culture for approximately 3 months it is possible that those mutations existed prior to the re-introduction of TgMSH1. This is consistent with the fact that 2 out of the 4 SNVs found in the complimented strains were also found in the MRC5 clones. To further test this idea, we maintained each of the WT, mutant and complemented strains for 8 passages (approximately 4 weeks) and re-sequenced each of the mtDNA fragments. No new SNVs were detected in the WT or any of the three complemented strains, but in contrast, two new SNVs were detected in MRC5a (Fig 1). Since after the 8 passages we did not re-clone, we were in effect sequencing from a population and likely only detecting dominant SNVs. It is important to note that the accumulation of SNVs in the MRC5 mutant and derived clones appears to be limited to the mtDNA as no variations were detected in either a 417-base pair fragment of nuclear DNA or a 1128 base pairs fragment of apicoplast DNA in any of the clones and strains sequenced. Furthermore, the SNVs detected are not ones seen within the fragments of these genes that are found throughout the nuclear DNA.

**Fig 2:**
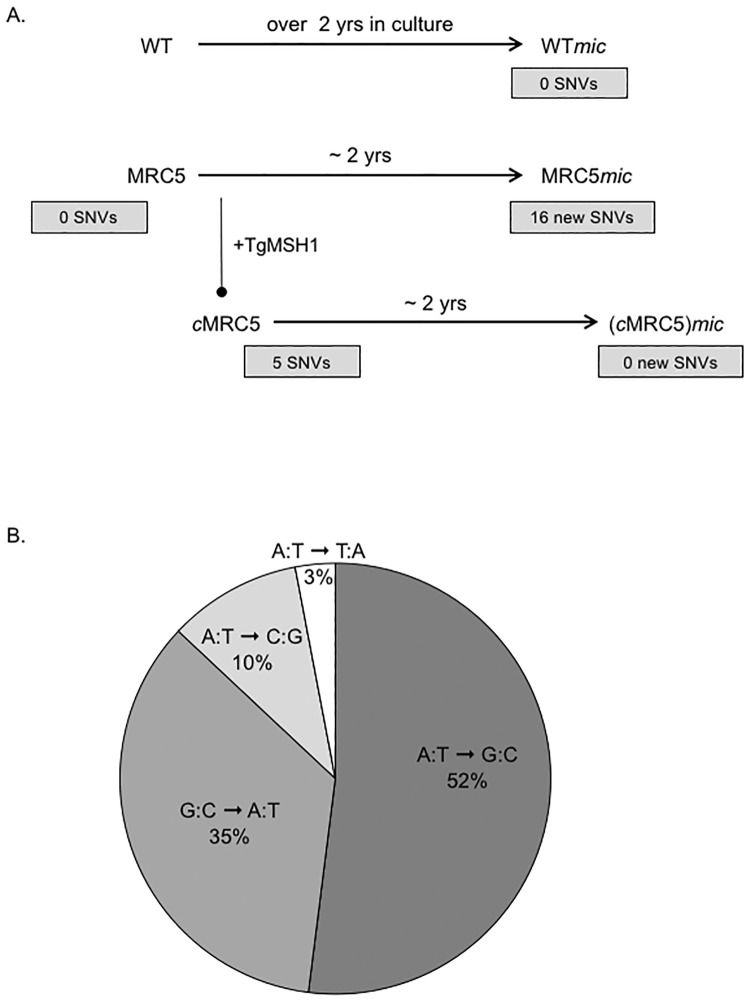
Sequencing of cytochrome b in parental, Tgmsh1 mutant and complemented strain after two years of culture. A. Total DNA was collected from parental, mutant and complemented strains before (WT, MRC5 and *c*MRC5) and after approximately 2 years of constant culture passage (WT*mic*, MRC5*mic* and (*c*MRC5)*mic*, where mic stands for “maintained in culture”). The entire coding region of the cytochrome b gene was amplified and sequenced. The number of single nucleotide variations identified is shown in the gray boxes below each strain. Arrows represent passage in culture over specified time. Line with black circle, indicates addition of TgMSH1 to generate the complemented MRC5 strain, *c*MRC5. B. Distribution of substitution types in the mitochondrial DNA of the *Tgmsh1* mutant strain. All 29 single nucleotide variations detected in the *Cox1*, *Cox3*, and *Cyb* fragments sequenced (Figs 1 and 2a) were cataloged and categorized based on the type of substitution.

To expand our analysis of mitochondrial DNA we took advantage that the sequence of the cytochrome b (*Cyb*) messenger RNA, which is 1,120 bases in length, has been previously determined and published [28]. Accordingly, we sequenced amplicons of the entire coding region of cytochrome b from the parental, mutant and complemented strains before (WT, MRC5 and *c*MRC5) and after approximately 2 years of constant culture passage (WT*mic*, MRC5*mic* and (*c*MRC5)*mic*, where mic stands for “maintained in culture”). While we do not detect any new SNVs within cytochrome b in either the parental or complemented strains after constant passage in culture for two years, we detected the emergence of 16 SNVs within cytochrome b in mutant MRC5 when comparing over the same period of time (Fig 2A and Table 1). Out of the 16 SNVs detected in *Cyb* in mutant strain MRC5, 8 generate missense mutation and 8 are silent (Table 1). As we directly sequenced the *Cyb* amplicon it is not known whether all SNVs are present simultaneously within the mutant parasites. Nonetheless, these results suggest that absence of TgMSH1 results in accumulation of mutations in the mitochondrial DNA.

**Table 1:** 

Nucleotide	Protein	MRC5mic	*c*MRC5	*c*MRC5mic	Nucleotide	Protein	MRC5mic	*c*MRC5	*c*MRC5mic
A103G	I35V	x			T651G	C217W	x		
G154A	A52T	x			A659G	Y220C	x		
G168A	n.c.		x	x	T701C	M234T		x	x
T207C	n.c.	x			C757T	H253Y*	x		
A228G	n.c.		x	x	A758G	H253R*	x		
A286G	n.c.		x	x	A771G	n.c.	x		
G346A	A116T	x			A786G	n.c.	x		
T399C	n.c.	x			A846G	n.c.	x		
A567G	n.c.	x			T891C	n.c.	x		
G586A	A196T	x	x	x	T901C	n.c.	x		

List of nucleotide variations found within cytochrome b in the MCRC5 strain maintained in culture for prolonged time (MRC5mic), the complemented MRC5 strain (*c*MRC5), and the complemented strain maintained in culture (*c*MRC5mic). Generation of the various strains listed is illustrated in Fig 2. Nucleotide and amino acid numbers are based on the published cytochrome b sequence [28]. Marks (x) indicate variation is found in the particular strain listed on top of column. n.c. (no change) indicates the variation causes a silent mutation.

*If nucleotide mutation is CA757-758TG protein change is H253C.

The particular base changes observed in mutated DNA can provide clues as to the type of DNA damage incurred by the cell. Accordingly, we cataloged and categorized all the SNVs detected in mtDNA in strains lacking TgMSH1 (Fig 2B). Transitions accounted for the vast majority of substitutions (87%), with 52% of all SNVs being due to AT to GC changes and 35% due to GC to AT changes (Fig 2B). By contrast, transversions accounted for only 13% of all mutations detected in the *Tgmsh1* mutant strains (AT to TA 3% and AT to CG 10%, Fig 2B). This is consistent with what has been reported for yeast mitochondria, in which mismatch repair preferentially corrects transitions, while transversions are corrected through proofreading by the mitochondrial DNA (mtDNA) polymerase [29].

### Amount of mitochondrial DNA is affected by lack of TgMSH1

**Fig 3:**
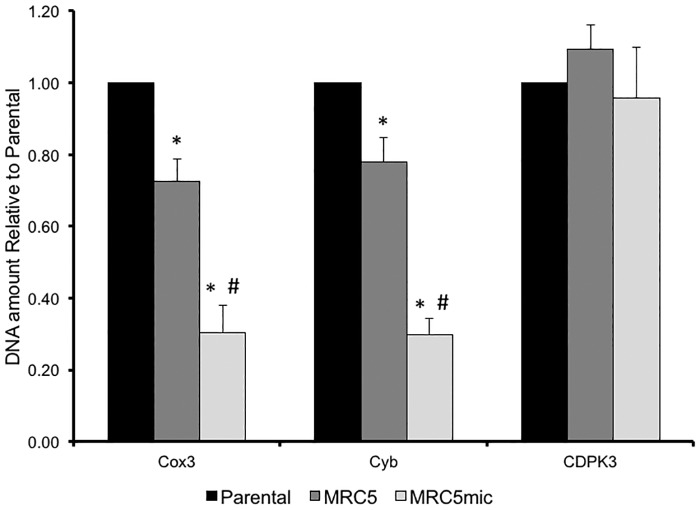
Quantification of mitochondrial DNA in parental and mutant strains. Quantitative PCR analysis was performed on genomic DNA obtained from parental, *Tgmsh1* mutant (MRC5) and Tgmsh1 maintained in constant culture (MRC5*mic*). Bar graph represents the relative copy number of *Cox1*, *Cyb* and CDPK3 (nuclear control) present in each strain as compared to the parental strain. Total DNA content was normalized using the *Toxoplasm*a alpha tubulin gene as a control. Graph represents three biological replicates; error bars are the standard deviation. Asterisks denote statistically different from parental based on one-way ANOVA followed by post-hoc Tukey HSD test (p<0.01). Pound sign denotes that MRC5mic is statistically different from MRC5 based on the same analysis (p<0.01).

Mitochondrially localized MutS homologs are known to function in maintaining the stability and integrity of the multi-copy mitochondrial genome in both yeast [17,30] and plants [20]. Accordingly, we investigated whether the copy number of mtDNA was affected in the TgMSH1 mutant strain (MRC5) using quantitative PCR (qPCR). To probe for mitochondrial DNA, we used primers specific to *Cox3* and *Cyb*, and used amplification of nuclear encoded tubulin to normalize across strains. The assay was validated by comparing nuclear DNA content across strains using primers against the single copy gene *TgCDPK3* [31], which as expected remained unchanged across all strains tested (Fig 3). By contrast, both mtDNA fragments assayed were present in less relative amounts in the initial MRC5 mutant strain and the same strain maintained in constant culture for approximately 2 years (Fig 3). Importantly, we noted that the relative amount of mtDNA decreases as the mutant strain is maintained in culture for prolonged periods of time, regardless of whether we probed for *Cox3* or *Cyb* (Fig 3). In the case of *Cox3*, for instance, the mtDNA amount is 72% of that in parental for the low passage MRC5 strain but drops to 30% of parental for the same strain after two years of passage. Thus, TgMSH1 appears to be critical for maintenance of the mitochondrial genome in *Toxoplasma*.

### Parasites lacking TgMSH1 function are tolerant to atovaquone treatment

**Fig 4:**
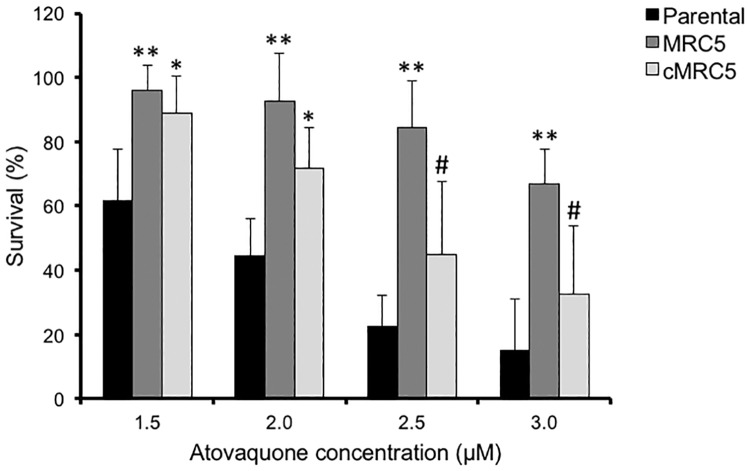
Sensitivity to atovaquone treatment. Parasites of wildtype, MRC5 mutant, or MRC5 complemented strains were allowed to form plaques in media containing atovaquone or solvent control. Percent survival represents the number of plaques formed in the presence of atovaquone after 12 days of growth divided by the number of plaques formed in the presence of solvent control. Data bars represent the average of four independent experiments, and error bars denote the standard deviation. Asterisks denote statistically different from parental based on one-way ANOVA followed by post-hoc Tukey HSD test (* p<0.05, ** p<0.01). Pound sign denotes that cMRC5 is statistically different from MRC5 based on the same analysis (#p<0.05).

Resistance to atovaquone in both *Toxoplasma* and *P*. *falciparum* is associated with point mutations in cytochrome B that prevent atovaquone from binding to the active site [28]. None of the mtDNA mutations we identified in the *Cyb* of the MRC5 mutant matched previously characterized point mutations linked to atovaquone resistance in *Toxoplasma* or *P*. *falciparum* [11,14–16]. Nonetheless, as we sequenced PCR fragments from uncloned parasites, it is plausible that some parasites within the culture of the *Tgmsh1* mutant strain carry mutations that could impart atovaquone resistance. To investigate this possibility, we seeded 10^6^ parasites of the parental or MRC5 strain into tissue culture flasks containing confluent human foreskin fibroblasts (HFFs, approximate multiplicity of infection of 0.2), in the presence of 3μM atovaquone. After five days, the flasks were fixed and stained with crystal violet to visualize plaques, which are clearings of the cell monolayer resulting from dividing parasites. While no plaques were detected in the flask containing parental parasites, we counted approximately 100 plaques in the flask seeded with the MRC5 mutant strain. Nonetheless, these plaques were much smaller in size than those formed by untreated parasites after five days. Furthermore, inspection at high magnification revealed a multitude of clusters of vacuoles that had not formed plaques but appeared to be viable. By contrast, neither plaques nor normal looking vacuoles were detected in the culture of 10^6^ atovaquone-treated parental strain parasites. This phenomenon suggested that all or a vast majority of MRC5 parasites added to the culture were surviving atovaquone treatment, but with a slow growth rate. To quantify this observation, we inoculated HFFs grown in 12-well tissue culture plates with 500 parasites of either the parental, MRC5, or complemented strain and allowed the parasites to grow in varying concentrations of atovaquone until plaques were detected. No plaques were detectable in any of the drug treated cultures at either 5 or 7 days, at which time-points large plaques are clearly visible in untreated parasites. Nonetheless, plaques were clearly visible for the mutant strain in the presence of atovaquone at 12 days. Accordingly, we fixed cultures at 12 days and enumerated plaques to compare survival among strains. In the presence of atovaquone we saw increased survival in the MRC5 mutant as compared to the parental or the complemented strains. At the highest concentration of atovaquone (3μM) 65% of the parasites survived while only 15% of the parental had survived (Fig 4). Interestingly, we only saw a partial rescue of the atovaquone sensitivity in the complemented strain (33% at 3μM), suggesting that the tolerance to atovaquone in the mutant strain could be due to irreversible events that had occurred prior to the addition of the wildtype copy of the gene.

It is plausible that during our 12-day assay to test atovaquone sensitivity, SNVs that would give rise to atovaquone resistance had emerged, and that in effect our assay artificially selected for atovaquone resistant mutants. This is highly unlikely, given that we see close to 100% survival at lethal concentrations, meaning that resistance would have had to develop within each of the plaques. Nonetheless, we tested this possibility by collecting parasites after 12 days of growth in 3μM atovaquone and testing their sensitivity to atovaquone through plaque assays (S2 Fig). We noted no difference in atovaquone sensitivity between the mutant parasites before and after the 12 days of growth in atovaquone. Thus, the ability of MRC5 parasites to survive atovaquone treatment is not due to an artificial selection within the 12 days of the assay.

### TgMSH1 mutant strain remains sensitive to inhibition of electron transport by atovaquone

**Fig 5:**
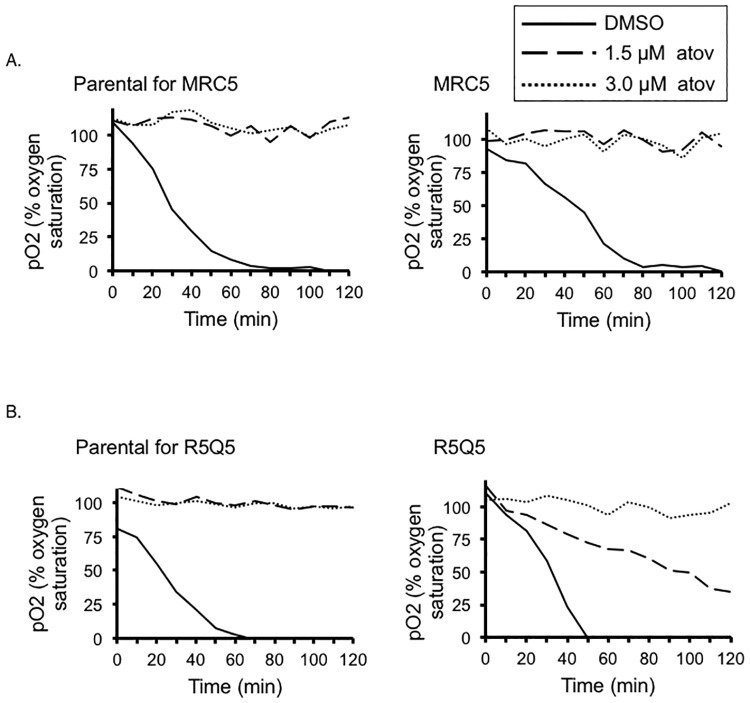
Effect of atovaquone treatment on respiration in the *Tgmsh1* mutant strain. Fifty millions of freshly harvested parental or *Tgmsh1* (MRC5) mutant parasites were added to Oxoplate (PreSens) wells containing artificial intracellular salt solution (AISS) buffer and drug for a final concentration of 1.5μM atovaquone (dashed line), 3.0μM atovaquone (dotted line) or DMSO vehicle control (solid line). Fluorescence readings at 540nm/650nm (indicator) and 540nm/590nm (reference) were measured every 10 minutes over the course of two hours at 37°C. Percent oxygen concentrations were calculated using the fluorescent readings obtained from each sample well and control wells (100% oxygen saturated solution or oxygen depleted solution) and then normalized to the internal reference fluorescence for each well. A representative graph of three biological replicates is shown, each data point is an average of at least three experimental repeats. B. The same experiment as described above was performed using the previously characterized atovaquone resistant strain R5Q5 [9] and its parental strain PDS.

The fact that we observe nearly 100% survival of the mutant strain at 1.5, 2.0 and 2.5 μM atovaquone (100%, 96%, 84%, and 67% respectively), all concentrations to which the parental strain shows sensitivity, would indicate that all of the mutant parasites that were added to the culture were already resistant to the drug. Atovaquone inhibits respiration but resistant strains are known to respire in the presence of the drug [6,32,33]. Accordingly, to test whether the MSH1 mutant strain was resistant to the effects of atovaquone on respiration, we tested oxygen consumption using commercially available oxygen detection plates. Specifically, parasites were added to a well of an OxoPlate (PreSens) containing an artificial intracellular salt solution buffer (AISS) and either vehicle control or atovaquone (1.5μM or 3.0μM). Changes in oxygen saturation were measured every ten minutes over a period of two hours at 37°C (Fig 5). As expected, both the wildtype and MRC5 mutant strains consumed oxygen in the presence of DMSO which was used as the solvent. Interestingly, however, respiration was inhibited in both the wildtype and the MRC5 mutant in the presence of atovaquone (Fig 5A). This would suggest that atovaquone was still able to inhibit respiration in MRC5 mutant, although they are able to survive prolonged atovaquone treatment in culture. To validate our approach, we measured the respiration of the atovaquone resistant parasite line, R5Q5, which contains a specific mutation in CYB that prevents atovaquone binding to the Q_o_ pocket, alongside it’s parental strain, PDS [28]. As predicted, R5Q5 continues to consume oxygen at 1.5μM, while it’s parental, PDS, does not (Fig 5B). It is important to note that the atovaquone IC_50_ for the PDS strain is 0.05uM, which is significantly lower than that for the RH derived strains, such as MRC5 [28]. These results confirm that the cytochrome b mutations found in the MRC5 mutant are not blocking atovaquone binding to its active site and that the ability of the mutant to grow in atovaquone is due to a novel mechanism.

### TgMSH1 mutant strain shows tolerance to other mitochondrial function inhibitors

**Fig 6:**
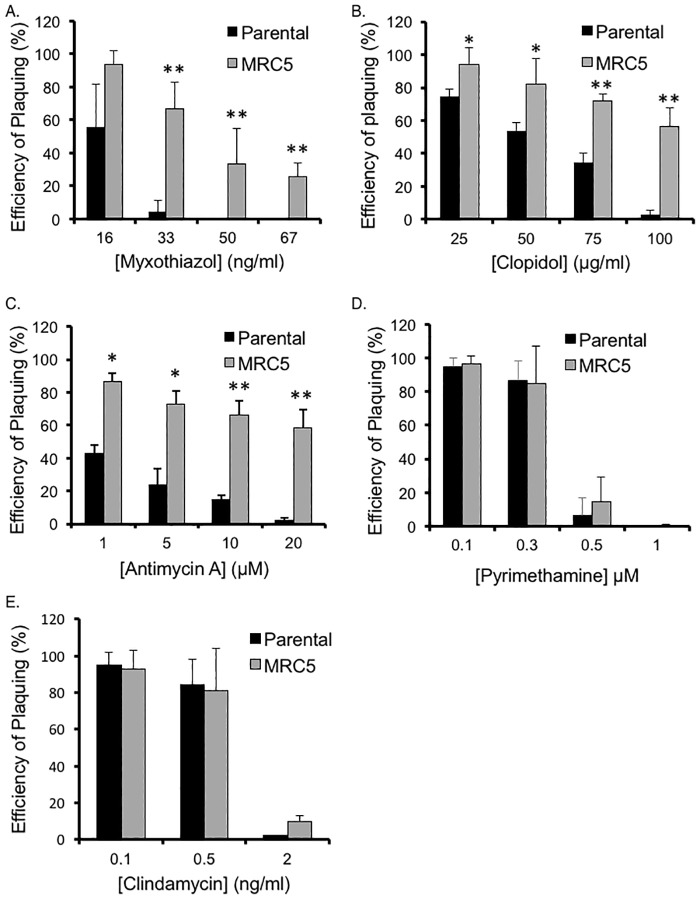
Sensitivity of *Tgmsh1* mutant strain to mitochondrial inhibitors. Parasites of the parental and MRC5 mutant strains were allowed to form plaques in presence of varying concentrations of myxothiazol (A), clopidol (B), antimycin A (C), pyrimethamine (D), or clindamycin (E). Percent survival represents the number of plaques formed in the presence of drug after 12 days of growth divided by the number of plaques formed in the presence of solvent. Data bars represent the average of three independent experiments, and error bars denote the standard deviation. Asterisks indicates significant statistical difference based on one-way ANOVA (*p<0.05, **p<0.005).

Resistance to atovaquone despite of effective inhibition of electron transport, as we see with our mutant, is reminiscent to what has been reported for the *P*. *falciparum* drug selected strain SB1-A6 [34]. Interestingly, SB1-A6 does not have mutations in cy*b* and is resistant not only to atovaquone but also to other cyt *bc*_1_ inhibitors such as stigmatellin, myxothiazol, antimycin A, clopidol, and WR243251 [34]. Accordingly, we tested whether *Toxoplasma* mutant strain MRC5 was also resistant or tolerant to three other electron transfer inhibitors: myxothiazol, clopidol, and antimycin A (Fig 6). Similar to what was noted with atovaquone, no obvious plaques were seen after 5 days of culture in presence of lethal doses of any of these three drugs with either the parental or mutant strain. Nonetheless, by 12 days of drug treatment we noted significant differences in sensitivity, with mutant MRC5 exhibiting high level of survival at normally lethal concentrations of myxothiazol, clopidol and antimycin A (Fig 6A–6C). For the MRC5 mutant we detect 57% survival at 100 μg/ml clopidol (vs 3% for parental strain), 58% at 20 μM antimycin A (vs 2% for parental), and 67% at 33 ng/ml myxothiazol (vs 4% for parental). Importantly, this multidrug tolerance appears to be specific to inhibitors of mitochondrial function as we do not see any differences between the strains in sensitivity to pyrimethamine, which inhibits dihydrofolate reductase [35–37], or clindamycin, which targets *Toxoplasma*’s apicoplast by binding to its ribosomal RNA [38], regardless of how long we incubate the parasites in the drug (Fig 6D and 6E).

### Growth of TgMSH1 mutant in atovaquone gives rise to strains with high level of drug resistance

**Fig 7:**
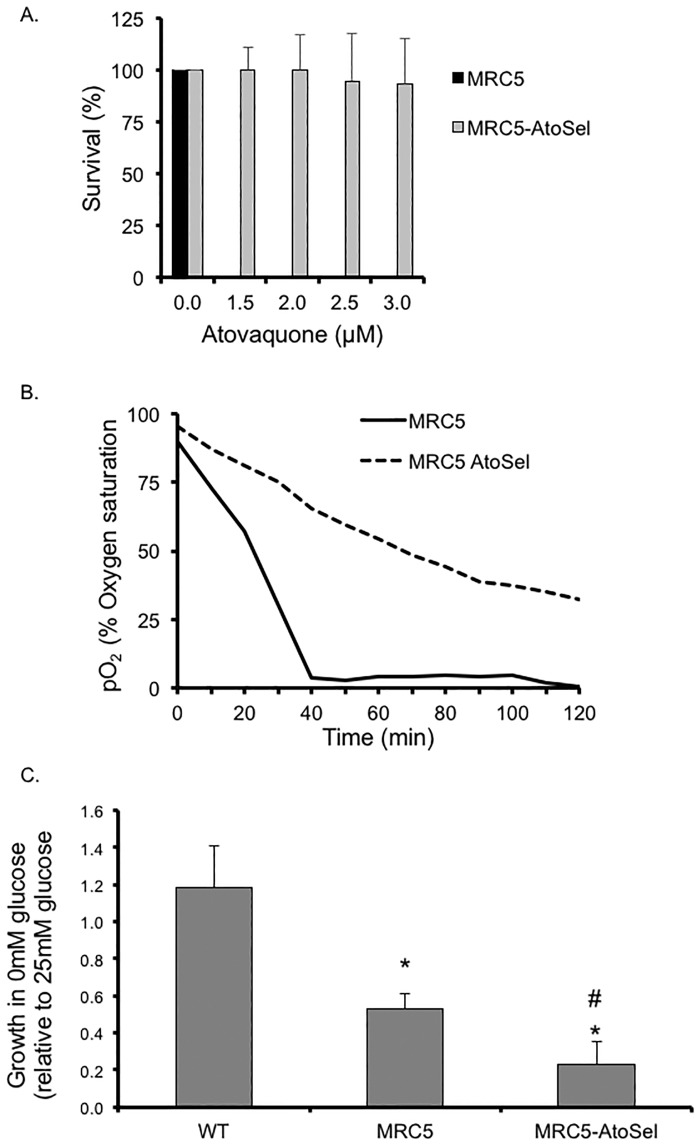
Characterization of *Tgmsh1* mutant parasites after selection in atovaquone. A. Intracellular MRC5 mutant parasites and MRC5 parasites selected for growth in atovaquone (MRC5-AtovSel) were allowed to form plaques in media containing atovaquone or solvent control. Percent survival represents the number of plaques formed in the presence of atovaquone after 5 days of growth as compared to the number of plaques formed in solvent control. Data bars represent the average of three independent experiments, and error bars denote the standard deviation. B. The ability of MRC5 (dashed line) and MRC5-AtoSel (solid line) parasites to consume oxygen under standard conditions was measured every 10 minutes over 120 minutes, as for Fig 5. Curves are average of triplicate data points. C. Parental, MRC5, and MRC5-AtoSel parasites were allowed to grow in either 0mM or 25mM glucose (normal culturing conditions) for five days. Level of parasite growth was determined based on β-galactosidase activity by reading absorbance at 24 hours following the addition of the substrate CPRG. For each strain, the growth rate in 0mM glucose was determined by dividing the measured absorbance level in 0mM glucose by that detected in 25mM glucose. Asterisk denotes statistically different from parental based on one-way ANOVA followed by post-hoc Tukey HSD Test (* p<0.01). Pound sign denotes that MRC5-AtoSel is statistically different from MRC5 based on the same analysis (# p<0.05).

Although we did not detect mutations that have been previously associated with atovaquone resistance in the TgMSH1 knockout strain, we hypothesize that, given its mutator phenotype, under the right pressure those mutations could emerge. Accordingly, we grew parental, MRC5 mutant and complemented parasites in continuous passage under the presence of 2.5μM atovaquone. Neither the parental or complemented parasites survived the treatment beyond three passages in three independent attempts, however the MRC5 mutant parasites were successfully maintained in atovaquone for approximately 30 passages, at which point we began seeing robust growth in the presence of the drug. The surviving parasites population were cloned through limiting dilution. To confirm and quantify the observed resistance we tested the ability of parasites to form plaques after 5 days of growth in the presence of atovaquone (Fig 7A). As expected, no plaques were detected in the parental or the original MRC5 mutant, however we see significant survival in the Atovaquone-selected MRC5 mutant (MRC5-AtoSel). At 3μM atovaquone we observe 86% (±2.87%) survival for the MRC5-AtoSel clone, while no plaques were detected in neither the parental nor the original MRC5 mutant at the same concentration (Fig 7A). Thus, continuous growth in atovaquone enhanced the ability of MRC5 to grow in atovaquone, suggesting the development of true resistance to the drug. To determine if the resistance was due to a mutation in Cyb we sequenced the gene and detected two new missense mutations in comparison to the MRC5 strain: R70K and A116V. Nonetheless, the affected amino acids have not been previously implicated with resistance and are not part of the atovaquone binding pocket, suggesting that there is an alternative atovaquone site in Cyb or, more likely, that the resistance phenotype could be due to a Cyb independent mechanism.

### Atovaquone selected mutants have significantly reduced respiration and are dependent on glucose

To explore the mechanism of resistance of the MRC5-AtoSel clone, we tested its ability to consume oxygen in the presence of atovaquone. Interestingly, we detected reduced oxygen consumption in these parasites in the absence of the inhibitor (Fig 7B). While we maintain the MRC5-AtoSel strain in atovaquone, parasites were grown in the absence of drug for 36 hours prior to the oxygen consumption assay. Thus, the lack of respiration is not likely due to a residual effect from the drug. Regardless, it appears that the MRC5-AtoSel strain can grow effectively despite a significant reduction respiration rate.

Given the reduced cellular respiration activity of the atovaquone-selected parasites, it would follow that these parasites are dependent on glycolysis as their primary source of cellular energy. To explore this possibility we grew parental, *Tgmsh1* mutant strain (MRC5), and MRC5-AtoSel parasites in media containing either 0 or 25 mM glucose (the amount present in the normal growth media used for parasite maintenance). Parasites of the parental strain exhibited the same level of growth with or without glucose (Fig 7C). By contrast, the MRC5-AtoSel parasites exhibited an approximately 80% reduction of growth in media lacking glucose as compared to normal media (Fig 7C). This indicates that this parasite strain is dependent on glycolysis due to impaired or inhibited mitochondrial function. Interestingly, the original MRC5 strain also exhibited reduced growth in the absence of glucose, albeit to a lesser extent than the atovaquone selected clone (Fig 7C).

### Atovaquone resistance reverts when selection pressure is removed

**Fig 8:**
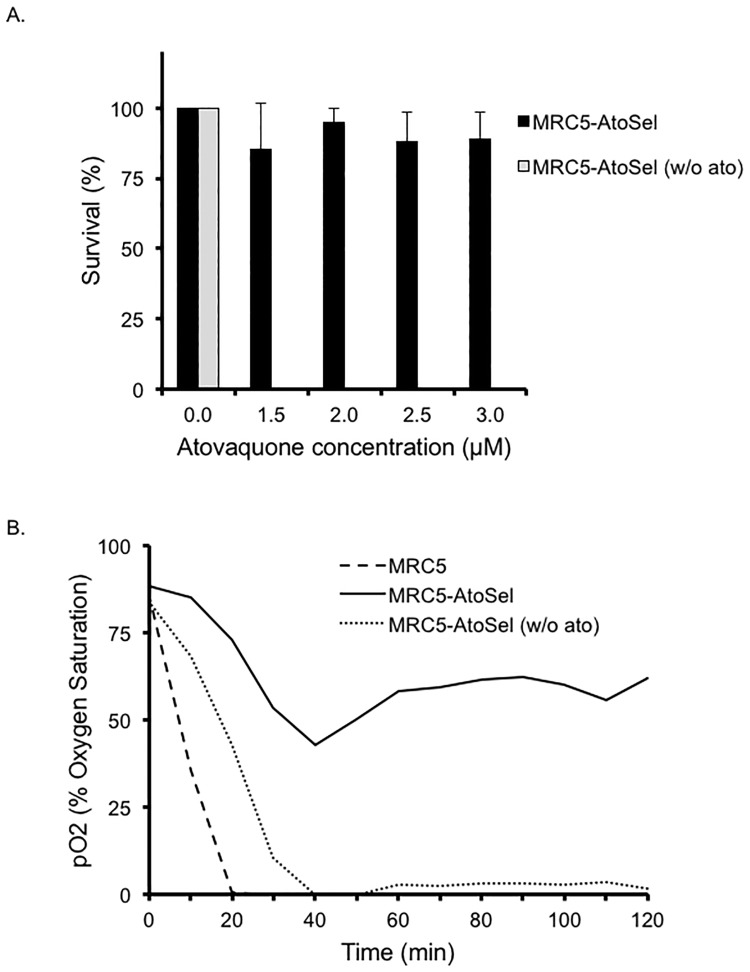
Atovaquone sensitivity of MRC5-AtovSel parasites maintained with and without atovaquone. A. The efficiency of plaque formation in atovaquone within five days was determined for MRC5 atovaquone selected parasites that were maintained in atovaquone (MRC5-AtovSel) and those that were maintained in the absence of atovaquone after the selection (MRC5-AtovSelRev). Percent survival represents the number of plaques formed in the presence of atovaquone after 5 days of growth divided by the number of plaques formed in the presence of solvent control, multiplied by 100. Data bars represent the average of three independent experiments, and error bars denote the standard deviation. B. The ability of MRC5 (dashed line), MRC5-AtoSel (solid line) and MRC5-AtovSelRev (dotted line) parasites to consume oxygen under standard conditions was measured every 10 minutes over 120 minutes as for Fig 6.

Interested in determining if the atovaquone resistance phenotype is stable, we removed the atovaquone selection pressure for approximately 30 passages (~3 months) and tested atovaquone sensitivity in comparison to the other strains. The atovaquone selected parasites, which were maintained in the presence of the inhibitor, continue to exhibit survival in atovaquone (Fig 8A). However, the MRC5AtoSel parasites for which selective pressure was removed (aka MRC5AtoSel w/o ato) were unable to form plaques following five days in the presence of atovaquone (Fig 8A). Thus, the apparent resistance to atovaquone of the MRC5AtoSel parasites is not stable and therefore unlikely to have a genetic basis. Additionally, we performed respiration assays with the MRC5 parasites, both atovaquone-selected clones, and the reverted parasites. Interestingly, while the atovaquone selected parasites have reduced respiration as noted before, the reverted parasites consume oxygen at higher rate (Fig 8B).

## Discussion

The requirement for monitoring and maintaining the fidelity of DNA in the mitochondria is underscored by the presence of various repair pathways within this organelle. Presumably, repair of errors or damage in mitochondrial DNA would be of particular importance in organisms that possess only a single mitochondrion, like the human pathogens, *Plasmodium falciparum* and *Toxoplasma gondii*. Surprisingly, very little is known about the proteins and pathways involved in maintaining and repairing mitochondrial DNA (mtDNA) in apicomplexan parasites. We have shown that genetic disruption of a MutS homolog protein in *Toxoplasma* (TgMSH1) leads to the accumulation of nucleotide variations in the mtDNA and loss of mtDNA. The functions of the *Toxoplasma* TgMSH1 described here, parallel those of plant and yeast MSH1. In addition, we noted that parasites lacking TgMSH1 have tolerance to diverse inhibitors of mitochondrial electron transfer and can adapt to growth in atovaquone, a potent cytochrome b inhibitor.

The nuclear encoded MutS homolog from *Saccharomyces cerevisiae*, ScMSH1, which preferentially binds to DNA mismatches and unpaired nucleotides *in vitro*, also localizes solely to the mitochondria [19]. Genetic disruption of yeast MSH1 results in the accumulation of point mutations in mitochondrial DNA [19] and instability of mtDNA [17,39]. Interestingly, analysis of different yeast MSH1 mutants indicate that besides mismatch repair, this protein might be involved in recombination surveillance and heteroduplex rejection [30]. Furthermore, it has been proposed that ScMSH1 plays several roles in mitochondrial base excision repair [40]. A similar role in mitochondrial DNA stability and organization has also been ascribed to the plant MSH1. Plant MSH1 regulates substoichiometric shifting [20,41], a phenomenon of that can produce novel subgenomic DNA molecules [42,43]. Thus, the two phenotypes observed with our TgMSH1 mutant strain, accumulation of single nucleotide mutations and loss of signal from PCR of mitochondrial DNA, are consistent with the functions of mitochondrial MutS homologs.

Whether *Toxoplasma* mitochondrial DNA undergoes substiochiometric shifting is not known, although it is plausible giving the parallels between the MSH1 from plants and *Toxoplasma*. In addition to the domains found in all eukaryotic MutS homologs, the plant MSH1 contains a GIY-YIG homing endonuclease not found in MutS homologs from yeast and mammalian cells [27]. Interestingly, MSH1 from *Toxoplasma* and related parasites of the phylum Apicomplexa contain this endonuclease domain. Nonetheless, detection of any possible restructuring of *Toxoplasma’s* mitochondrial DNA is complicated by the fact that its sequence and structure has not been completely deciphered. On the other hand, the mitochondrial DNA has been well characterized in various *Plasmodium* species and determined to be a 6 kb unit with tandemly arranged repeating sequences [44–50]. For *Plasmodium* parasites, the mitochondrial genome appears to be a multi-copy element with an estimated 150 per cell in *P*. *yoelii* [46] and 15 to 10 in *P*. *gallinaceum* [51]. Thus, it is likely that the mitochondrial DNA of *Toxoplasma* is multi-copy and composed of repeating elements. Indeed, our data shows variability in the amount of DNA detected for several fragments of mtDNA between strains (Fig 3). This result would indicate that the mitochondrial DNA is either multi-copy or repetitive and that TgMSH1 is required for its maintenance. At this point we do not know whether the reduction in quantitative PCR signal for mtDNA fragments in MRC5 is due to a loss of copies of a presumably multi-copy genome or recombination and rearrangements within the existing genomes.

Both loss of genetic material and accumulation of single nucleotide mutations would be predicted to affect mitochondrial function if either were to affect the expression levels or sequence of the mitochondrially encoded proteins. Interestingly, the *Tgmsh1* mutant strain MRC5 exhibits reduced ability to grow in the absence of glucose, which would suggest an impairment of mitochondrial function as it relates to ATP production. As parasites lacking TgMSH1 do not exhibit a significant reduction in propagation efficiency under normal culture conditions [21], it would appear that the mutant parasites can adapt to compromised mitochondrial function. This adaptation might account for the mutant’s ability to grow in the presence of atovaquone. Atovaquone, a naphthoquinone, functions as a competitive inhibitor of the Qo site in complex III (cytochrome bc1) preventing ubiquinol (QH_2_) from completing the Q cycle and ultimately disrupting the electron flux through the rest of the electron transport chain. Atovaquone resistance in both *Toxoplasma* and *Plasmodium spp*. have been attributed to cytochrome b mutations that disrupt atovaquone binding to the Qo pocket [9,33]. Nonetheless, none of the missense mutations that we detect within Cyb in our mutant strains are in amino acids associated with resistance [6,9,33,52–54] or that are known to come in contact with atovaquone [55]. Accordingly, the mutant parasites remain sensitive to the effects of atovaquone on respiration despite their ability to grow in presence of the drug. Thus, it is likely that the ability of the mutant to survive atovaquone treatment is not directly due to the mutations in *Cyb*, but rather a consequence of a generalized adaptation to the loss of mitochondrial function.

The idea that the *Tgmsh1* mutant has adapted to disrupted mitochondrial function is supported by data showing that the mutant strain can survive not only in the presence of atovaquone, but also in myxothiazol, antimycin A and clopidol. Like atovaquone, myxothiazol targets the cytochrome bc1 complex by binding to the Qo site in cytochrome b, but likely within distinct binding regions. On the other hand, antimycin A targets the quinone reductase (Qi) site [56,57] while the target site for clopidol is less characterized [58]. The fact that the mutant parasite is resistant or tolerant to these distinct inhibitors further points to an alternative mechanism of resistance. Possible scenarios include a shift in metabolism that allows the parasites to survive inhibition of the electron transport chain, albeit at a lower replication rate, or an ability by the mutant parasite to remain quiescent until the drug concentration in the media is reduced by turn over. Attempts to differentiate between these two possibilities proved to be inconclusive. Nonetheless, we were able to obtain parasites that replicated robustly in atovaquone by continuous passage of the mutant strain in presence of the drug. These adapted strains remained sensitive to atovaquone in terms of inhibition of respiration and were more dependent on glucose than the original MRC5 mutants. The increased dependence on glucose for parasite growth would suggest that the *Tgmsh1* mutant strain had already begun shifting towards glycolysis as the primary source of energy, while the atovaquone selected parasites shifted even further to favor glycolysis. Interestingly, this enhanced tolerance to atovaquone is reversible, which underscores that it is not due to a genetic mutation. Thus, we hypothesize that, to overcome the disruption of mitochondrial DNA, the parasites lacking TgMSH1 have adapted their physiology as to survive with a functionally compromised mitochondrion.

Interestingly, tolerance to diverse cyt *bc1* complex inhibitors has been reported *for P*. *falciparum* strain SB1-A6. This strain was generated by culturing parasites in the presence of 3-(6,6,6-trifluorohexyloxy)-6-amino-acridone (6-NH2 Ac), a potent inhibitor of *P*. *falciparum* that is postulated to target *cyt b* [34,59]. Like our *Tgmsh1* mutant, SB1-A6 can grow in the presence of atovaquone, myxothiazol and antimycin A, even though these drugs effectively inhibit the electron transport chain [34]. Importantly, no mutations are present in the *Cyb* sequence of SB1-A6 suggesting a biochemical adaptation that allows the strain to bypass the need for conventional electron transport. It has been proposed that in *Plasmodium spp*. electron transport is not critical for energy production and that instead it is essential for de novo pyrimidine synthesis via dihydroorotate dehydrogenase (DHODH) [60]. This is unlikely to be the case for *Toxoplasma*, in which atovaquone inhibits mitochondrial function but does not affect pyrimidine biosynthesis [61]. Nonetheless, the fact that both *Toxoplasma* and *Plasmodium* can adapt as to survive the inhibition of mitochondrial function indicates a remarkable ability by these parasites to adapt their physiology. Whether the mechanisms responsible for such adaptation are the same in both *Plasmodium* and *Toxoplasma* is not currently known and would necessitate a more in-depth analysis of the metabolism and physiology of the mutant strains.

The analysis of our *Toxoplasma* mutant strain, MRC5, has revealed TgMSH1 as critical for the maintenance and fidelity of the mitochondrial DNA. Given how little is known about *Toxoplasma*’s mitochondrial genome, the identification of a protein that associates and acts on mitochondrial DNA could open the door to a more complete understanding of the apicomplexan mitochondrion. Future work would focus on dissecting the contribution of the various domains found in TgMSH1 to its function in DNA repair and identification of protein interactors. Furthermore, our results provide important information about atovaquone, showing that resistance to this clinically relevant drug can occur through mechanisms independent of mutations in the binding site of its target protein Cyb. *Toxoplasma*, like *Plasmodium*, appears to have the ability to forgo the need of the electron transport chain and adapt its biochemistry to survive treatment with diverse mitochondrial inhibitors. Understanding the mechanism behind this adaptation will undoubtedly reveal novel information about the physiology of this important human pathogen, which could educate the development of new treatments and avoid the development of drug resistance.

## Materials and methods

### Ethics statement

No animals or human subjects were used in this study.

### Parasite and host cell maintenance

For the experiments described here, we used MRC5 parasite line previously characterized [21] and its parental strain, RHΔhpt+GFP+βgal, which expresses GFP and β-galactosidase (βgal) [62]. *Toxoplasma* tachyzoites were propagated by passaging in human foreskin fibroblasts (HFFs, purchased from the American Tissue Culture Collection, ATCC) in a humidified incubator maintained at a temperature of 37°C and 5% CO_2_ concentration. Normal growth medium used was Dulbecco’s Modified Eagle Medium (Life Technologies) supplemented with 10% fetal bovine serum (Atlanta Biologicals), 2 mM L-gluatamine (Life Technologies) and 50 μg/mL penicillin-streptomycin (Life Technologies). Atovaquone was purchased from Sigma-Aldrich and prepared using DMSO as solvent.

### Sequencing of mitochondrial, nuclear and apicoplast DNA fragments

Total genomic DNA was isolated from freshly lysed out parasites using the DNeasy blood and tissue kit (Qiagen). Fragments of *Cox1*, *Cox3*, and *Cyb*, nuclear, and apicoplast DNA, were amplified using Phusion high fidelity polymerase and primers 1 to 10 listed in S1 Table (S1 Table). Full length *Cyb* was amplified using primers 11 and 12. Resulting DNA fragments were purified using gel or column purification kits from Qiagen, and submitted for sequencing at ACGT, INC (Wheeling, IL).

### Quantification mitochondrial DNA copy number

Total DNA was collected from freshly egressed parasites using the DNeasy blood and tissue kit (Qiagen). The mtDNA copy number was determined by real-time polymerase chain reaction using primers 3 and 4 for *Cox3* and 5 and 6 for *Cyb* (S1 Table) and following manufacturer’s instructions for the Fast SYBR Green Master Mix (Applied Biosystems, Thermo Fisher Scientific, Vilnius, Lithuania). As control for nuclear DNA we used primers 13 and 14 for CDPK3 (S1 Table). The quantity of DNA was normalized to the expression of the nuclear *Toxoplasma* alpha tubulin (TUBA1) gene using primers 15 and 16 (S1 Table). The baseline and threshold (Ct) was determined by the Sequence Detection System software imbedded into the Applied Biosystems program and verified manually. The relative amount of mtDNA was calculated using the ΔΔC_t_ method. Specifically: ΔΔC_t_ = Δ*CT*_(target sample)_ − Δ*CT*_(parental)_; where Δ*CT* = *CT*_(target gene)_−*CT*_(TUBA1)_. Finally, the relative DNA amount was expressed as 2^ΔΔ*CT*^.

### Quantification of drug resistance

Drug resistance was quantified by assessing each strain’s ability to form plaques in the presence of the drug tested. Five hundred parasites of each strain were added to HFFs grown to confluency in normal culture conditions in 12 well tissue culture plates. Following a 4–5 hours incubation period, media and uninvaded parasites were aspirated and media containing the drug at various concentrations were added to the wells. For the no drug control, we used DMSO, or appropriate solvent, at the highest amount used. Parasites were incubated under these conditions until plaques formed, after which the cultures were fixed with 100% methanol, stained with crystal violet, and plaques were counted using a stereomicroscope. The percent survival, based on the ability of parasites to form plaques, was calculated as the number of plaques formed in drug divided by the number of plaques formed in vehicle control, and multiplied by 100. Statistical significance was tested using one-way Anova followed by post-hoc Tukey HSD test as well as Bonderroni and Holm multiple comparison using Prism.

### Oxygen consumption assays

To measure parasite respiration, we used OxoPlate (OP96F) from PreSens according to the manufacturer’s instructions. In brief, intracellular parasites were passed through a syringe, filtered through a 3 μM to eliminate host cell debris, and resuspended at 5 x 10^8^ p/ml in an artificial intracellular salt solution (AISS) [63]. A total of 5 x 10^7^ parasites were added to each well containing either vehicle control or atovaquone at 2x (2.0μM or 6.0μM) in AISS buffer. Changes in oxygen saturation were measured every ten minutes over a period of two hours at 37°C using a Biotek Synergy H1 fluorescence plate reader set at 540/650 nm (indicator) and 540/590 nm (reference); integration time: 0 to 500μs and gain 50. Wells containing fully saturated water or oxygen depleted water (with sodium sulfite) were used for calibration. Calculations to determine the percent oxygen saturation in the well were carried out as outlined in the manual.

### Generation of MRC5AtoSelected and reverted parasites

Parental and MRC5 parasite clones were subjected to growth under 2.5μM atovaquone for 50 passages. As a control, the same clone was maintained without drug alongside the drug-selected strain. After 30 passages, the MRC5AtoSel parasite population was cloned out under limiting dilution. To obtain the reverted strain the atovaquone selection pressure was removed for 30 passages.

### Measuring parasite growth in no glucose conditions

Intracellular parasites were freed from cells by passing through a syringe after which they were passed through a 3μM filter to eliminate host cell debris and resuspended in complete DMEM media lacking phenol red to a concentration of 1000 parasites/ml. 100 parasites of each strain were added to HFFs grown in 96-well plates in phenol red free media. The parasites were allowed to invade for 2–3 hours in the presence of glucose, after which, the media was removed and the wells were washed twice with no glucose media. Finally, the wells were filled with phenol-red free media containing either 0 or 25mM glucose and the parasites were allowed to grow for five days under standard parasite growth conditions. Approximately 24 hours prior to measurements, Chlorophenol red-β-D-galactopyranoside, (CPRG, Sigma) was added to a final concentration of 100 μM [64]. Endpoint absorbance measurements were taken at 540 nM wavelength using a Synergy H1 Plate Reader. Control values from wells containing uninfected cells were obtained and subtracted from the experimental absorbance measurements. Finally, the corrected absorbance values were normalized by dividing by the values of parasites grown in normal glucose (25 mM). The experiment was repeated for a total of three times statistical significance was tested as described above for quantification of drug resistance.

## Supporting information

S1 Fig Sequence of mitochondrial DNA fragments. Shown are the sequences of PCR fragments used to monitor Cox I, Cox III and Cyb in parental and mutant strains. Underlined regions indicate primers used to amplify fragments. Highlighted bases are ones found to be mutated in the *Tgmsh1* mutant parasites.
(PDF)

S2 Fig Atovaquone sensitivity of MRC5 parasites grown in atovaquone for 12 days. Either parental strain or MRC5 parasites that were recovered after 12 days of growth in atovaquone were grown in no or 3 μM atovaquone for either 5 days or 12 days and cultures were fixed and stained to reveal plaques. Image of a representative plaque assay is shown. Despite growth in the presence of atovaquone for 12 days, MRC5 parasites do not form plaques by five days indicating that we have not selected for resistant parasites within the 12 day period used for the assay shown in Fig 5.
(PDF)

S1 Table Sequence of all primers used in this study. (PDF)
