# Supplementary material for: Lack of mitochondrial MutS homolog 1 in Toxoplasma gondii disrupts maintenance and fidelity of mitochondrial DNA and reveals metabolic plasticity
Source: PLoS One. 2017 Nov 15;12(11):e0188040. doi: 10.1371/journal.pone.0188040 (PMC5687708; doi:10.1371/journal.pone.0188040)
Supplement: S1 Fig — Shown are the sequences of PCR fragments used to monitor Cox I, Cox III and Cyb in parental and mutant strains. Underlined regions indicate primers used to amplify fragments. Highlighted bases are ones found to be mutated in the Tgmsh1 mutant parasites. (PDF) [file pone.0188040.s001.pdf]

S1 Fig

Cox I fragment

TGATTGGTTAATTGGAGGACTTGCTGTTCTTGGTATCAGTAGTATCC
TTAGTTCTATTAACTTCCTTGGTACATGTGTCTTTATGGGATCTTGT
GCAGGAGCAAAAAATTATATTTTATATATTTGGTCTATTATTTTTAC
AGCTCTTATGCTAGTATTTACACTACCTATTTTAACAGGTGGACTAG
TTATGATCTTATTAGATTTACATGTAAATACAGAATTTTATGATTCT
ATGTATTCTGGTGATAGTGTCTTATATCAACATCTATTCTGGTTTTT
TGGACATCCAGAAGTATATATTCTAATTCTACCTGCTTTTGGTGTTG
TATCTCAAAC

Cox III fragment

TCATGTTATTGTCGGTGCTATCTTGGGTTTCTTTAATCAGGGTATGT
ATAGCTCTCTAGTTACATATTTACCAGTAAACTGCATAACTTTGAGT
AAATGCAAAGGTACATTATGTAAAATCTTCTCAGAACCATTTACAAT
CTTATATCTACATTTCGTCGAAGCAGTGTGGATAATGATC

Cyb fragment

CGTAGTAACCTCCAAGTAGCCAAGGTACCAAATATGGTATTGGAGAA
AGGAGGTTTGTAATGACTGTAGCACCCCAGAAACTCATTTGTCCCCA
TGGTAGTACATATCCGAGGAAGGCAGTGGCTATAGTAAGTAAATATA
AAACTAAACCAGACATCCAAGCGGTAGTT
