# Supplementary figures and images for: Lack of mitochondrial MutS homolog 1 in Toxoplasma gondii disrupts maintenance and fidelity of mitochondrial DNA and reveals metabolic plasticity
Source: PLoS One. 2017 Nov 15;12(11):e0188040. doi: 10.1371/journal.pone.0188040 (PMC5687708; doi:10.1371/journal.pone.0188040)

S2 Fig

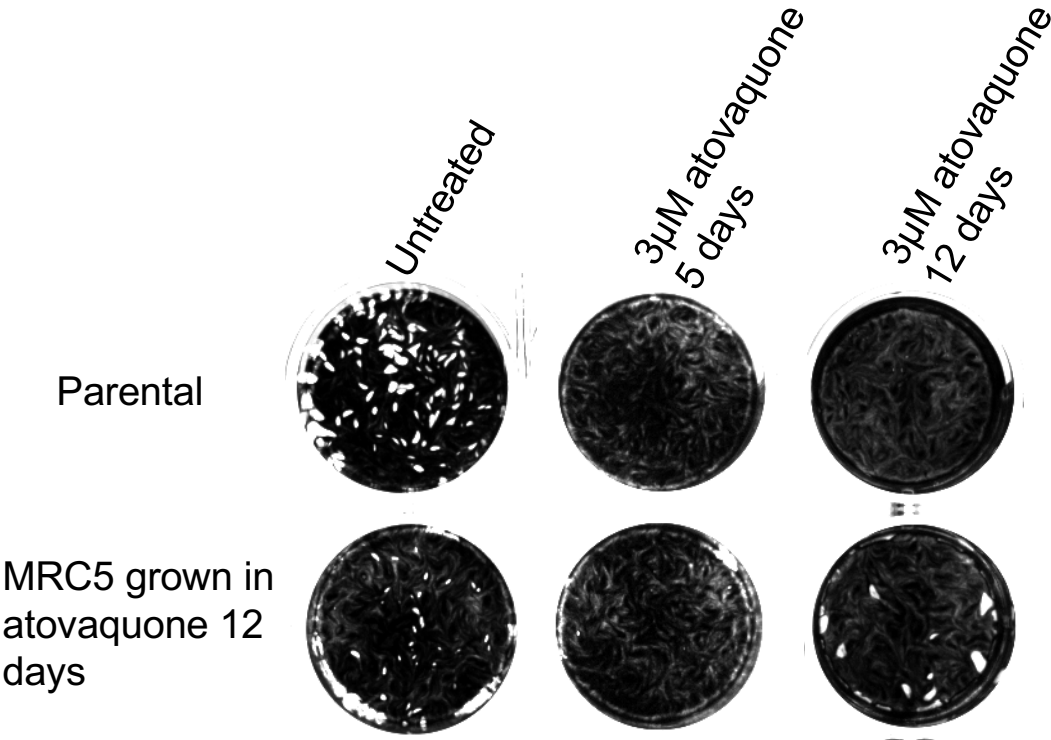

Supplement: S2 Fig — Either parental strain or MRC5 parasites that were recovered after 12 days of growth in atovaquone were grown in no or 3 μM atovaquone for either 5 days or 12 days and cultures were fixed and stained to reveal plaques. Image of a representative plaque assay is shown. Despite growth in the presence of atovaquone for 12 days, MRC5 parasites do not form plaques by five days indicating that we have not selected for resistant parasites within the 12 day period used for the assay shown in Fig 5. (PDF) [file pone.0188040.s002.pdf]
