# Supplementary material for: Lack of mitochondrial MutS homolog 1 in Toxoplasma gondii disrupts maintenance and fidelity of mitochondrial DNA and reveals metabolic plasticity
Source: PLoS One. 2017 Nov 15;12(11):e0188040. doi: 10.1371/journal.pone.0188040 (PMC5687708; doi:10.1371/journal.pone.0188040)
Supplement: S1 Table — (PDF) [file pone.0188040.s003.pdf]

S1 Table. Sequence of all primers used in this study

Use	Primer #	Name	Primer Sequence
Amplification of cytochrome c oxidase I (cox1) for sequencing	1	cox1 Forward	TGATTGGTTAATTGGAGGACTTGCTGT
	2	cox1 Reverse	GTTTGAGATACAACACCAAAAGCAGG
Amplification of cytochrome c oxidase III (cox3) for sequencing and qPCR	3	cox3 Forward	TCATGTTATTGTCGGTGCTATCTTGG
	4	cox3 Reverse	GATCATTATCCACACTGCTTCGACGA
Amplification of apocytochrome b (cob) for sequencing and qPCR	5	cob Forward	CGTAGTAACCTCCAAGTAGCCAAGG
	6	cob Reverse	AACTACCGCTTGGATGTCTGGTTTAG
Amplification of nuclear DNA fragment for sequencing	7	Sag1 3'UTR	GGCCCGGTACCTCACCGTTGTGCTCACTTCTC
	8	Sag1 3'UTR	GGCCCGAGCTCCCCCTCGGGGGGGCAAGAATTGTGTTAAC
Amplification of Plastid DNA for sequencing	9	RRFL Forward	TATGCCCGTACTAAAACTGACACA
	10	RRFL Reverse	CCTACTTATATGCTTTCAGTAGTTATT
Amplification of entire CYB gene for sequencing	11	MSL Forward	CGGAATTCATGAGTCTATTCCGGGCACA
	12	QAN Reverse	CGGGATCCGTTTGCTTGGAAGCTGTAGT
Amplification of CDPK3 fragment for quantitative PCR	13	qCDPK3 Forward	CTCCTCGGTACACCTCCATG
	14	qCDPK3 Reverse	GGTAAGTGGTGCCAGAGAGC
Amplification of Tubulin A1 for normalization of quantitative PCR	15	qTubA1 Forward	GCATGATCAGCAACAGCACT
	16	qTubA1 Reverse	GAGAGCAGCCAAATCCTCAC
